# From mechanistic promise to clinical translation: Passive heat therapy as a cardiovascular and functional adjunct in ageing and disease

**DOI:** 10.1113/EP093511

**Published:** 2025-11-29

**Authors:** Fergus K. O'Connor, Surendran Sabapathy, Aaron J. E. Bach, Bryce N. Balmain, Llion Roberts, Norman R. Morris

**Affiliations:** ^1^ School of Allied Health, Sport and Social Work Griffith University Gold Coast QLD Australia; ^2^ Australian Centre for Precision Health and Technology Griffith University Gold Coast Queensland Australia; ^3^ Griffith Institute for Human and Environmental Resilience Griffith University Gold Coast Queensland Australia; ^4^ Allied Health Research Collaborative, The Prince Charles Hospital Metro North Hospital and Health Service Brisbane Queensland Australia; ^5^ Institute for Exercise and Environmental Medicine, Texas Health Presbyterian Hospital Dallas, Department of Internal Medicine University of Texas Southwestern Medical Center Dallas Texas USA

**Keywords:** cardiovascular rehabilitation, functional capacity, peripheral perfusion

We read with great interest the Viewpoint by Brocherie et al. (2025) entitled *‘*Turning up the heat: Can thermal therapy really protect muscle health in older adults?’ Their thoughtful synthesis highlights the physiological rationale through which localised or systemic heating might preserve muscle function, including enhanced calcium kinetics, increased rate of force development and potential activation of angiogenic pathways. We wish to extend this discussion by providing recent evidence that supports and broadens these mechanistic insights, while positioning a translational pathway forward.

## 1 TRANSLATING MECHANISTIC GAINS TO FUNCTIONAL OUTCOMES

In support of the enhanced skeletal muscle contractile function following passive heating discussed by Brocherie et al. (2025), our recent randomised crossover trial provides complementary, translational evidence of the potential benefits of passive heating (O'Connor et al., 2025). Specifically, our data show that a single 45‐min bout of lower‐limb (knee‐down) heating (42°C) in older adults (median [Q1, Q3] age: 71 [67, 74] years) with heart failure with reduced ejection fraction (HFrEF) significantly increased exercise endurance, as measured by the endurance shuttle‐walk test, immediately following the immersion protocol. The improved endurance capacity was accompanied by increases in femoral artery blood flow and tissue oxygenation of the gastrocnemius (O'Connor et al., 2025). Beyond mechanistic improvements in muscle force generation (Denny et al., 2025), these findings demonstrate that passive heating can acutely enhance functional exercise capacity, extending the view that contractile performance in aged muscle can be improved with such an intervention (Brocherie et al., 2025). Importantly, these benefits occurred without adverse haemodynamic responses, underscoring the potential feasibility and safety of well‐controlled, localised heating, even in a higher‐risk clinical population (O'Connor et al., 2025).

## 2 SYSTEMIC AND ADAPTIVE BENEFITS

### 2.1 Passive heat therapy as a standalone intervention

As Brocherie et al. (2025) state, the potential benefits of passive heating may extend beyond acute functional gains. While long‐term effects on muscle health remain under‐explored, recent systematic reviews (Price et al., 2025; Rodrigues et al., 2025) report significant cardiovascular improvements following passive heat therapy interventions in both healthy and clinical populations, even in the absence of exercise. Emerging evidence also suggests that passive heating may provide some cardiometabolic benefits, albeit data supporting this notion are limited (Price et al., 2025). Collectively, these data align with the hypothesis advanced by Brocherie et al. (2025) that repeated thermal stress induced by passive heating can activate hormetic pathways conferring both metabolic and vascular resilience.

### 2.2 Passive heat therapy as an adjunct to exercise training programmes

Although the chronic cardiovascular benefits of passive heat therapy are becoming increasingly evident (Price et al., 2025; Rodrigues et al., 2025), whether the acute benefits following single‐bout passive heating (Denny et al., 2025; O'Connor et al., 2025) translate into improved work capacity during subsequent (i.e. immediately following passive heating) exercise training sessions remains unknown. If this notion is proven true, it stands to reason that passive heat therapy has the potential to enhance adaptation from standard exercise training protocols. Thus, passive heat therapy presents as a promising, low‐cost and (potentially) accessible adjunct to standard exercise training. This may be particularly appealing for individuals unable to achieve sufficient training intensity or volume due to age, disease or deconditioning. Further trials are needed to clarify optimal integration strategies, and delivery modalities of passive heating to quantify additive benefits to exercise‐induced adaptation.

## 3 FROM MECHANISM TO APPLICATION: TOWARD INTEGRATED EXERCISE TRAINING PROGRAMMES

While further evidence stemming from laboratory‐based investigations is likely still required, we concur with Brocherie et al. (2025) that the integration of passive heat therapy in practice demands individualised, controlled and monitored application. Realising this outcome will depend on a deliberate and collaborative translation pathway. Meaningful integration into clinical exercise settings requires direct engagement with participants and clinicians to co‐design heating protocols that are practical, acceptable and responsive to the needs of those undergoing rehabilitation. This participatory process ensures that the intervention evolves within, rather than adjacent to, existing rehabilitation workflows, promoting sustained adoption, patient confidence, and clinician buy‐in. Only once these outcomes have been achieved, and further safety interventions (e.g. sensor‐based safeguards analogous to falls‐monitoring devices) have been designed and developed should the case for home‐based passive heat therapy be confidently advanced.

Building on this foundation, the physiological rationale for pre‐exercise heating becomes clear, whereby transient improvements in peripheral perfusion, tissue oxygenation and contractile characteristics may allow individuals to perform more work or achieve comparable workloads with less effort. Repeated exposure, even in the absence of exercise, could promote physiological adaptations, such as enhanced aerobic capacity, vascular and endothelial function, muscular strength, improving prognostic outcomes. Finally, an additional, unexplored benefit of passive heat therapy could be its utility as a viable alternative to standard exercise‐based rehabilitation. In this context, passive heat therapy may enable individuals who were otherwise unwilling or unable to undertake structured exercise to obtain the non‐exercise associated benefits of clinical rehabilitation. Thus, passive heat therapy presents as an accessible, low‐barrier adjunct that has the potential to augment both participation and physiological adaptation within, and alongside structured exercise programmes, bridging the gap between laboratory efficacy and real‐world clinical benefit.

## 4 CONCLUSION

Taken together, a growing body of evidence (Denny et al., 2025; O'Connor et al., 2025; Price et al., 2025; Rodrigues et al., 2025) is positioning passive heat therapy as a promising, low‐cost and accessible strategy to enhance acute exercise performance, while providing additional cardiovascular benefit when used in chronic applications. As the authors aptly suggest, ‘turning up the heat’ may indeed help preserve function across the lifespan, provided it is done safely, systematically and with clinical intent.

## AUTHOR CONTRIBUTIONS

All authors have read and approved the final version of this manuscript and agree to be accountable for all aspects of the work in ensuring that questions related to the accuracy or integrity of any part of the work are appropriately investigated and resolved. All persons designated as authors qualify for authorship, and all those who qualify for authorship are listed.

## CONFLICT OF INTEREST

None declared.

## FUNDING INFORMATION

None.
